# Restoring Cognitive Functions Using Non-Invasive Brain Stimulation Techniques in Patients with Cerebellar Disorders

**DOI:** 10.3389/fpsyt.2014.00033

**Published:** 2014-04-02

**Authors:** Paul A. Pope, R. Chris Miall

**Affiliations:** ^1^School of Psychology, University of Birmingham, Birmingham, UK

**Keywords:** tDCS, TMS, cerebellar cognitive affective syndrome, cognitive rehabilitation, spinocerebellar degeneration

## Abstract

Numerous studies have highlighted the possibility of modulating the excitability of cerebro–cerebellar circuits bi-directionally using transcranial electrical brain stimulation, in a manner akin to that observed using magnetic stimulation protocols. It has been proposed that cerebellar stimulation activates Purkinje cells in the cerebellar cortex, leading to inhibition of the dentate nucleus, which exerts a tonic facilitatory drive onto motor and cognitive regions of cortex through a synaptic relay in the ventral–lateral thalamus. Some cerebellar deficits present with cognitive impairments if damage to non-motor regions of the cerebellum disrupts the coupling with cerebral cortical areas for thinking and reasoning. Indeed, white matter changes in the dentato–rubral tract correlate with cognitive assessments in patients with Friedreich ataxia, suggesting that this pathway is one component of the anatomical substrate supporting a cerebellar contribution to cognition. An understanding of the physiology of the cerebro–cerebellar pathway previously helped us to constrain our interpretation of results from two recent studies in which we showed cognitive enhancements in healthy participants during tests of arithmetic after electrical stimulation of the cerebellum, but only when task demands were high. Others studies have also shown how excitation of the prefrontal cortex can enhance performance in a variety of working memory tasks. Thus, future efforts might be guided toward neuro-enhancement in certain patient populations, using what is commonly termed “non-invasive brain stimulation” as a cognitive rehabilitation tool to modulate cerebro–cerebellar circuits, or for stimulation over the cerebral cortex to compensate for decreased cerebellar drive to this region. This article will address these possibilities with a review of the relevant literature covering ataxias and cerebellar cognitive affective disorders, which are characterized by thalamo–cortical disturbances.

## Introduction

Clinicians have been directly exciting the cerebellar cortex with implanted electrodes in epileptic patients and in those with schizophrenia and depression since the 1970s with good therapeutic results (1), demonstrating the use of constant electrical stimulation for the treatment of behavioral disorders and epilepsy. Today, transcranial brain stimulation techniques [often referred to as non-invasive brain stimulation (NIBS)], such as repetitive transcranial magnetic stimulation (rTMS) and transcranial direct current stimulation (tDCS), are realized to have the capacity to systematically modify behavior by inducing lasting changes in underlying brain functions, and are useful approaches to studying brain–behavior relationships in healthy participants. They have also been used to study mechanisms of cortical plasticity, and both techniques have been implicated as therapeutic tools for the treatment of motor and cognitive deficits in patients after stroke, and in cerebellar disease (2, 3). In recent years, cerebellar-tDCS has grown in popularity in various laboratories and clinics, partly because the lateral cerebellar hemispheres, which are thought to be involved in cognition, are most accessible to transcranial electrical stimulation, are sensitive to the effects of polarizing currents, and because the procedure is relatively inexpensive and easy to perform.

### Mechanisms of action and effects of tDCS

The mechanisms of action and effects of tDCS on the human cerebellum are inferred from animal studies, or from indirect effects on motor cortex, and from modeling data. In humans, the procedure typically involves delivering 1–2 mA of DC stimulation through a pair of saline-soaked electrodes: one stimulation electrode on scalp overlying the cerebellum, and the other reference electrode on the ipsilateral head or shoulder. Intracerebral current flow between the two electrodes has relatively little functional spread to neighboring regions [e.g., visual cortex (4)] and is thought to excite or depress Purkinje cells in the cerebellar cortex, producing both neurophysiological and behavioral changes. The effects are polarity-specific as evidenced by the consequences of cerebellar stimulation on motor cortex excitability (5). Anodal stimulation has an excitatory effect and increases the output of Purkinje cells; increasing inhibition of the facilitatory pathway from the cerebellar nuclei to cerebral cortex. Cathodal stimulation has an opposite effect, i.e., dis-inhibition of the cerebral cortex by reducing Purkinje cell inhibition of the cerebellar nuclei. However, the after-effects of TMS (6) and tDCS (7) over motor cortex are highly variable between individuals, and not always polarity-specific, which highlights the need to better understand individual factors that determine the efficacy of NIBS (e.g., neural excitability and/or cognitive capacity) and to develop improved protocols for delivering brain stimulation. Effects of stimulation are also different depending on whether behavior is tested during (on-line effects) or after (off-line effects) the stimulation period, which typically last 15–20 min, suggesting that on-line effects may include changes in ion concentration gradients and cell membrane potentials, while off-line effects might include longer lasting changes in neural activity due to altered intracellular processes (e.g., receptor plasticity). Polarity-specific effects on cognitive functions are more difficult to detect and to interpret than the direct effects of the cerebellum on motor areas due to cerebellar-brain inhibition (CBI). Nonetheless, anatomical studies in primates reveal how Purkinje cells could exert a facilitatory drive onto both motor and cognitive circuits, via a synaptic relay in the ventral–lateral thalamus (8). And, associative plasticity induced by sensory/motor stimuli paired at 25 ms – paired associative stimulation (PAS), can be blocked by cerebellar-tDCS, demonstrating how the cerebellum can exert a remote influence over excitability in the cerebral cortex (9). Thus, changes in both motor and cognitive functions are physiologically plausible via electrical stimulation of the cerebello–thalamo–cortical pathway.

### tDCS after-effects and the cerebellum

Polarizing the brain with cortical scalp electrodes as treatment for remedying cognitive deficits in human participants is not new. In the 1960s, Lippold and colleagues demonstrated beneficial effects in certain psychiatric disorders caused by long duration stimulation (up to 10 h) at small current strengths over the forehead (10, 11). The authors were able to distinguish positive and negative polarization effects on mood in the majority of cases. Scalp-positive effects included an increase in the patients’ involvement with the environment (e.g., alertness and cheerfulness), and scalp-negative effects included environmental inhibition and withdrawal (e.g., quietness). Due to a recent revival in this method, there is now a better understanding of tDCS-induced effects and evidence that cerebellar-tDCS can modulate, and in some cases, enhance cognitive functions and behavioral performance in healthy participants [reviewed in Ref. (12, 13)]. For example, in 2005, Ferrucci and colleagues measured off-line tDCS effects during a modified version of the Sternberg item recognition task (i.e., identifying the presence or absence of a digit from a list of previously presented visual items after a memory maintenance period) in healthy participants (14). Fifteen minutes of cerebellar stimulation (irrespective of electrical polarity or activity of visual cortex) impaired the usual practice-dependent proficiency increase associated with this task. Five years later, this result was reproduced by Boehringer et al. (15). While neither study found tDCS to enhance performance, the work by Boehringer and colleagues did demonstrate that tDCS could alter performance during visual item recognition as a function of task difficulty or when cognitive load is set at a specific level. These studies show how tDCS can alter cerebellar cognitive functions, and hint toward situations where tDCS is most efficient.

**Figure 1:**
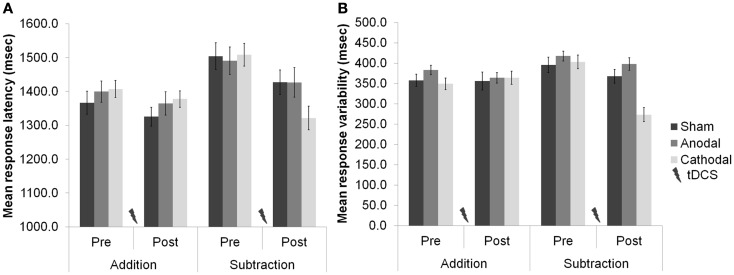
**(A)** Response latencies (mean + 1 SEM, *n* = 20) selectively improved after cerebellar cathodal stimulation from session one (pre-stimulation) to session two (post-stimulation), significantly more in the subtraction task than in the addition task. **(B)** The variability of participants’ responses also selectively improved significantly between sessions during subtraction, but not during addition. Modified from Pope and Miall (16).

Task difficulty was a major feature of our recent study of cerebellar functioning during tests of verbal working memory [WM; (16)], in which we applied tDCS over the right cerebellar hemisphere and showed neuro-enhancement during a demanding subtraction version of a mental arithmetic task [the paced auditory serial subtraction task (PASST)] with high cognitive load, but not during a simpler and less demanding addition version [the paced auditory serial addition task (PASAT)]. In short, cathodal stimulation improved task accuracy, response speed, and response variability [relative to anodal and sham stimulation (see Figure 1)]. As both tasks share similar motor control (i.e., verbal operations), but dissimilar cognitive load (i.e., mental effort), we speculated that cathodal depression of the right cerebellar cortex might release additional cognitive resources required when demands are high by dis-inhibition of the left prefrontal cortex to which it projects via the cerebello–thalamo–cortical pathway (3). Supporting this view, and the emergent role for the cerebellum in cognition and emotion (17, 18), is the finding that functional connectivity between the cerebellum and prefrontal cortex during mathematics is task- and difficulty-sensitive (19). This result was demonstrated shortly after MR signal coherence measures were first used to detect cerebellar–prefrontal and cerebellar–parietal connections (20), lending further support to the idea that the cerebellum can influence cognitive processes in the prefrontal cortex: a major site for many WM operations.

**Figure 2:**
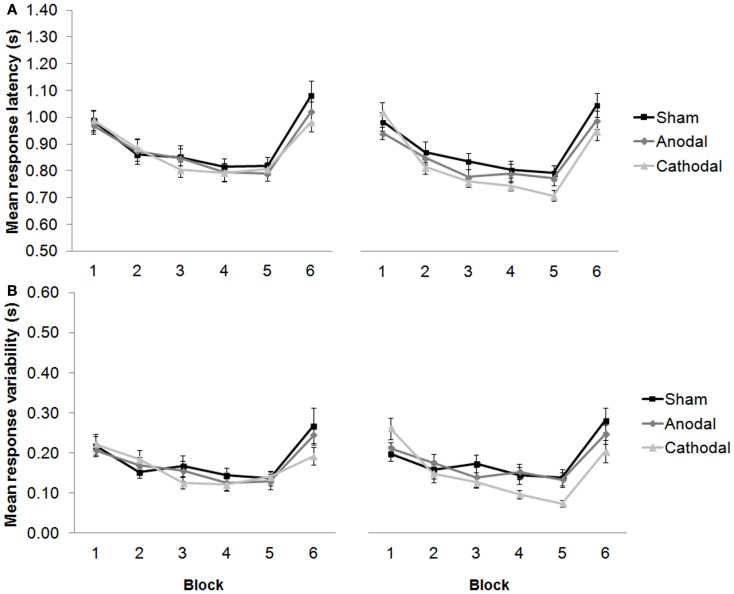
**(A)** Response latencies (mean + 1 SEM, *n* = 20) decreased across repeated presentation of the same sets of noun–verb pairs between blocks 1–5 (new words were presented in block 6), and selectively improved after cathodal stimulation from session one [pre-stimulation (left panel)] to session two [post-stimulation (right panel)]. **(B)** The variability of participants’ responses also selectively improved significantly between blocks 1–5, and between sessions. Modified from Pope and Miall (16).

Interestingly, our 2012 work predicts that anodal stimulation over the left dorsolateral prefrontal cortex (DLPFC) should selectively improve performance during subtraction, but not addition task versions. Indeed, others have shown how stimulating the DLPFC can enhance arithmetic performance over long durations, improve neurovascular coupling (21), and facilitate solution generation for difficult problems, but not for easy problems (22). In fact, electrical stimulation of the prefrontal cortex can enhance performance in a variety of WM tasks in healthy participants (23, 24), leading researchers to employ tDCS as a therapeutic tool for the treatment of cognitive deficits in patients after stroke (25), and in patients with Parkinson’s disease (26). In our cerebellar-tDCS study (16), we also employed a language protocol as well as the arithmetic study, and observed responses that got faster over five consecutive blocks of trials in which participants generated verbs in response to visually presented nouns (see Figure 2). This priming effect complements the results of our arithmetic study, and also findings by others showing how anodal tDCS over the left DLPFC can improve verbal fluency (27) and picture naming latencies (28). It supports the hypothesis that the same facilitation patterns may be observed after cathodal tDCS over the right cerebellar hemisphere as can be seen by anodal stimulation over the frontal cortex. Taken together, these findings support a role for the cerebellum – albeit indirect – in language, learning, and memory (29).

### tDCS influences cerebro–cerebellar connectivity

**Figure 3:**
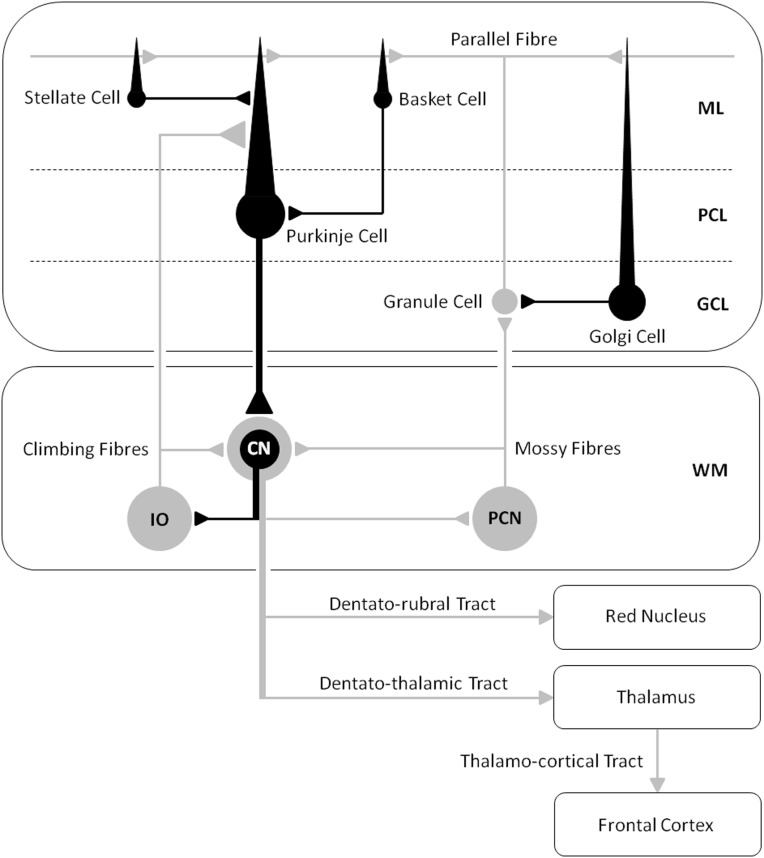
**A schematic diagram of the main circuits and interneurones in the cerebellar cortex and the principal white matter tracts connecting the cerebellum to the cerebrum – cerebro–cerebellar connectivity [after Voogd and Glickstein (31); redrawn by authors]**. Inhibitory cells/synapses are shown in black, excitatory cells/synapses are shown in gray. ML, molecular layer; PCL, Purkinje cell layer; GCL, granule cell layer; WM, white matter layer; CN, cerebellar nuclei; IO, inferior olive; PCN, precerebellar neurones.

Our tDCS work (16) shows how an understanding of neuro-enhancements in healthy participants is firmly constrained by cognitive and anatomical hypotheses regarding WM capacity and cerebro–cerebellar connectivity, where cerebellar stimulation may most effectively modulate cognition and performance when participants fully engage in a task, or when the task maximally excites the cerebellar–cortical pathway. Indeed, an fMRI study by Salmi and colleagues previously showed how a load increase in cognition during WM tasks is associated with enhanced neural activity in both cerebral and cerebellar areas, which they suggested was involved with optimization of response speed (30). They also showed with MR tractography how crus I/II in the posterior lateral cerebellum was linked with the lateral prefrontal areas activated by an increase in cognitive load, whereas the anterior cerebellar lobe was not. Based on the available literature, cerebellar-tDCS could be expected to influence cognition during certain WM tasks via excitation of the cerebello–thalamo–cortical pathway (see Figure 3), leading researchers to speculate on the efficacy of this technique as a therapeutic tool for treating cognitive deficits in patients with cerebellar disease (3).

### NIBS and cerebellar ataxias

The cerebro–cerebellar circuits have been proposed as the anatomical substrate of the cerebellar involvement in executive functions, WM, and emotion in patients with the cerebellar cognitive affective syndrome [CCAS (17, 18)]. And the cerebellum’s role in cognition in the context of adaptation, expertise, and giftedness is said to accelerate information processing in WM and to make thinking and reasoning more efficient (32). Some ataxic patients can also present with cognitive and emotional impairments if damage to non-motor regions of the cerebellum disrupts the coupling with cerebral cortical areas for thinking and reasoning. In fact, white matter changes in the dentato–rubral tract [but not the dentato–thalamic or thalamo–cortical tracts (see Figure 3)] have been shown to correlate with cognitive assessment in patients with Friedreich’s ataxia (FA), suggesting that this pathway is an important contributor to cognitive impairments in this disease (33, 34). Genetically confirmed FA patients present with impairments in processing speed, conceptual thinking, verbal fluency, acquisition of verbal information, use of semantic strategies in retrieval, visuoperceptive and visuoconstructive functions, and action naming (35). However, DC stimulation of the cerebellar cortex may not at first hand prove effective in remedying the symptoms of this autosomal recessive inherited disease since cerebro–cerebellar circuits, including the dentato–rubral tract, become progressively damaged due to atrophy of the dentate nucleus – negating possible therapeutic benefits from tDCS or TMS. But this has not prevented clinicians from employing other interventions such as cognitive rehabilitation therapy (CRT), and physical and occupational therapy to help improve/stabilize cognition, mood and motor functions in patients with spinocerebellar degeneration, by asking them to carry-out a battery of cognitive tests and activities of daily living that after treatment changed the rate of disease progression in some patients (36, 37). Cerebellar-tDCS could then be used alongside rehabilitative interventions to provide a synergistic effect – further improving the management of these patients. Recently, tDCS applied as an adjunct to cognitive behavioral therapy (CBT) has been successful in treating depression in patients over the course of a year, whose symptoms were otherwise resistant to other forms of treatment (38). Such a study demonstrates a favorable synergistic interaction between two very different interventions, opening up new possibilities for the use of tDCS as a cognitive neuro-rehabilitation tool. It is also worth speculating whether the excitatory effects of tDCS alone could prove helpful in reducing cognitive decline/stabilizing mood and motor functions in cerebellar patients by exciting cerebro–cerebellar connectivity; preventing or slowing down further damage in analogy to the slowing of disease progression in patients with FA (36) and spinocerebellar ataxia (37). This might be expected if chronic cerebellar damage reduces the excitability of motor and visual cortices (18). Behavioral gains induced by tDCS do increase functional connectivity, for example after stroke (39). Thus, the tDCS enhances activity, and it seems, maintains the cerebello–thalamo–cortical pathway.

### Motor and cognitive improvements after cerebellar-TMS

Transcranial direct current stimulation has yet to be performed in patients with cerebellar ataxia with the aim of evaluating cognitive functions, although improved motor symptoms (reduced amplitude of upper limb stretch reflexes) have been reported in these patients after anodal stimulation of the cerebellum, which increased the inhibitory effect exerted by the cerebellar cortex upon the cerebellar nuclei (40). Anodal tDCS over the motor cortex can also improve gait symmetry in patients with cerebellar ataxia for a short-term period by restoring motor cortex activity deprived from the cerebro–cerebellar circuit (41). However, improved cognition in an ataxic patient has been demonstrated using TMS, which uses electromagnetic induction instead of a direct electrical current to activate the brain. In a case study by Farzan and colleagues (2), a patient with a diagnosis of idiopathic late-onset cerebellar atrophy with speech and gait difficulties underwent 21 daily sessions of TMS at maximum output over the cerebellum. After treatment, the authors observed improvements in the patients’ functional mobility (postural control and walking) and dual-tasking (naming supermarket items while walking). The therapeutic mechanisms were also investigated using dual-coil paired-pulse TMS to measure cerebello–thalamo–cortical connectivity, before and after treatment. The difference between treatments was marked by an increase (facilitation) in motor evoked potentials induced by motor cortical stimulation when the cerebellum was also excited a few milliseconds beforehand, demonstrating enhanced activity in contralateral motor cortex that reflected reduced CBI. The reduction in CBI lasted 6 months after treatment. The authors attributed the improvements in cognitive function to a consequence of enhanced motor function and liberation of resources for the performance of the dual-task, thus enabling the patient to name more items while walking with more ease. The TMS-induced reduction in CBI may also have improved prefrontal cortical function directly, through exciting cerebellar projections to this area, thus improving cognitive capacity. This second explanation compares well with that from our own work showing a selective improvement in verbal WM after cerebellar-tDCS (see above).

### NIBS and cerebellar cognitive affective disorders

The cerebro–cerebellar circuits that can be enhanced by TMS in cerebellar ataxic patients presenting with cognitive impairments are disrupted in patients that develop the CCAS following a lesion or damage to the non-motor cerebellum (17, 18). A defect associated with a lesion in one cerebellar hemisphere is decreased excitability of the contralateral prefrontal area. Thus, symptoms that are part of the CCAS extend beyond problems with motor control, co-ordination and balance, and include problems with executive functions, WM, linguistic performance, and changes in emotion and personality (18). The use of cerebellar-tDCS to enhance cognition via its remote neuromodulatory effect on prefrontal areas can be anticipated too on the basis of existing TMS studies of cerebellar cognitive functions. This should lead to improving mental flexibility (e.g., multi-tasking) in CCAS patients. For example, facilitatory effects of TMS have been observed during procedural learning [e.g., serial reaction time task (SRTT)], which involves acquiring a skill (beyond just motor control) through repeated performance and practice. This task is thought to involve connections between the cerebellum and the prefrontal cortex via the thalamus, and damage in any one of these regions is likely to impair performance. Indeed, TMS over the DLPFC interferes with procedural learning when applied over the hemisphere contralateral to the performing hand (42). A patient has also been studied with a left cerebellar lesion and a selective deficit in procedural learning, as evidenced by poor performance with the left hand on the SSRT (43). By decreasing cortical excitability of the right (unaffected) cerebellum or the left DLPFC (in separate sessions) with 1 Hz rTMS for 10 min, the deficit recovered and task performance markedly improved. Interestingly, inhibition of the right DLPFC or a control fronto-parietal region did not change the patient’s performance. It is interesting to speculate whether the inhibitory effects of cathodal tDCS of the same regions might produce similar results. Nonetheless, the authors explained these findings in terms of the modulation of a set of inhibitory and excitatory connections between the lateral cerebellar hemisphere and the contralateral prefrontal area induced by the inhibitory effect of TMS – restoring the balance of cortical activation. Trains of epidural anodal/cathodal DC stimulation over the cerebellum in rats has also shown how this structure can exert a remote neuromodulatory effect upon the excitability of the primary motor cortex – reshaping the representation of muscles on motor cortex (44). In an earlier paper, the same authors employed anodal tDCS to antagonize motor cortex hypoexcitability contralateral to a hemicerebellar ablation in rats, and they speculated that by setting the motor cortex at an appropriate level of excitability, tDCS might be used to modulate motor cortex excitability after acute cerebellar dysfunction (45). In humans, neuromodulatory effects from cerebellar stimulation have revealed how cathodal tDCS decreases CBI, in contrast with anodal tDCS that increases the cerebellum’s inhibitory tone over the motor cortex (5). Taken together, tDCS of the cerebellum and prefrontal cortex, either jointly or in separate sessions, might offer a new treatment for restoring the balance between these two regions, which normally work together to fine-tune behavior and optimize performance.

## Conclusion

Many studies involving healthy participants and certain patient populations demonstrate the value of NIBS as the technique of choice for producing plastic changes in the brain, and as a research tool for testing hypotheses about how motor and cognitive functions are performed and how cerebro–cerebellar circuits subserve these operations. Based on the available literature, we see five possible approaches to cognitive rehabilitation using NIBS in patients with damage at various sites in this circuit. (1) Cerebellar-tDCS could reduce cognitive decline and/or improve mood in ataxic patients. By increasing the excitability of cerebellar projections to areas of the prefrontal cortex, this may prevent further damage and decline of this pathway and potentially enhance functional connectivity. (2) NIBS could also be used as an adjunct to other types of therapy (e.g., CRT or CBT), improving their therapeutic efficacy when treating the decline of cerebellar cognitive functions. This is because evidence suggests that NIBS enhance the neuroplastic effects of adjunct non-stimulation therapy. And this may apply not only in diseases primarily involving the cerebellum, but also in those affecting interconnected regions where the cerebellum exerts a modulatory influence. (3) Enhancing the coupling between one side of the cerebellum and the contralateral region of frontal cortex is another possibility in which the facilitatory effects of NIBS could be exploited: improving cognitive capacity and motor control in patients with pure cerebellar ataxias. This would free up more cognitive resources for dual-tasking (e.g., talking whilst walking) – minimizing the risk of falls in aged cerebellar patients with cognitive decline. Even in healthy individuals, NIBS may be anticipated to improve motor and cognitive functions and enhance performance by boosting cerebro–cerebellar connectivity. A sedentary life does not engage this circuit much. Expert performers such as musicians (46) and athletes (47), have a significantly larger cerebellum than non-experts, suggesting that increased activity increases neural volume and probably neural connectivity. (4) The neuromodulatory effects of cerebellar stimulation might prove successful in restoring the balance of inhibitory and excitatory connections in the cerebrum, which can be dysfunctional in patients with cerebellar damage. Studies show that the normal effects of CBI, which typically decreases excitability of the motor cortex, are reduced or absent in patients with degeneration or lesions of the efferent system from the cerebellum (48, 49), confirming the clinical effectiveness of NIBS to manage motor deficits in cerebellar ataxias (40, 41). (5) Lastly, one can foresee a procedure that combines the inhibitory effects of cathodal tDCS or low frequency rTMS to decrease CBI, with the excitatory effects of anodal tDCS or high frequency rTMS to excite the DLPFC. This dual-site stimulation paradigm could be employed to enhance the dis-inhibition of the cerebral cortex, restoring system balance after cerebellar disease and permitting improved cognitive functions. However, the type of stimulation (e.g., inhibitory versus excitatory) and stimulation paradigm (e.g., single- versus dual-site) to be employed as part of an effective treatment plan will be governed by understanding each patients’ specific medical condition. Future research will likely explore these ideas and must be directed toward understanding individual factors that determine the efficacy of NIBS, leading to better procedures and protocols for delivering NIBS as a cognitive rehabilitation tool for neuro-enhancement.

## Conflict of Interest Statement

The authors declare that the research was conducted in the absence of any commercial or financial relationships that could be construed as a potential conflict of interest.
